# Parabolic, Flight-Induced, Acute Hypergravity and Microgravity Effects on the Beating Rate of Human Cardiomyocytes

**DOI:** 10.3390/cells8040352

**Published:** 2019-04-14

**Authors:** Aviseka Acharya, Sonja Brungs, Yannick Lichterfeld, Jürgen Hescheler, Ruth Hemmersbach, Helene Boeuf, Agapios Sachinidis

**Affiliations:** 1 Institute of Neurophysiology, Center for Molecular Medicine Cologne (CMMC), University of Cologne, 50931 Cologne, Germany; aacharya@uni-koeln.de (A.A.); j.hescheler@uni-koeln.de (J.H.); 2 German Aerospace Center, Institute of Aerospace Medicine, Gravitational Biology, Linder Hoehe, 51147 Cologne, Germany; Sonja.Brungs@dlr.de (S.B.); Yannick.Lichterfeld@dlr.de (Y.L.); Ruth.Hemmersbach@dlr.de (R.H.); 3 INSERM (French National Institute of Health and Medical Research), U1026-Biotis, Université de Bordeaux, 33076 Bordeaux, France; helene.boeuf@u-bordeaux.fr

**Keywords:** microgravity, hypergravity, human induced pluripotent stem cells, cardiomyocytes, adrenoceptor agonist, Isoprenaline, L-type Ca^2+^ channels, Bay-K8644

## Abstract

Functional studies of human induced pluripotent stem cell (hiPSC)-derived cardiomyocytes (hCMs) under different gravity conditions contribute to aerospace medical research. To study the effects of altered gravity on hCMs, we exposed them to acute hypergravity and microgravity phases in the presence and absence of the β-adrenoceptor isoprenalin (ISO), L-type Ca^2+^ channel (LTCC) agonist Bay-K8644, or LTCC blocker nifedipine, and monitored their beating rate (BR). These logistically demanding experiments were executed during the 66th Parabolic Flight Campaign of the European Space Agency. The hCM cultures were exposed to 31 alternating hypergravity, microgravity, and hypergravity phases, each lasting 20–22 s. During the parabolic flight experiment, BR and cell viability were monitored using the xCELLigence real-time cell analyzer Cardio Instrument^®^. Corresponding experiments were performed on the ground (1 *g*), using an identical set-up. Our results showed that BR continuously increased during the parabolic flight, reaching a 40% maximal increase after 15 parabolas, compared with the pre-parabolic (1 *g*) phase. However, in the presence of the LTCC blocker nifedipine, no change in BR was observed, even after 31 parabolas. We surmise that the parabola-mediated increase in BR was induced by the LTCC blocker. Moreover, the increase in BR induced by ISO and Bay-K8644 during the pre-parabola phase was further elevated by 20% after 25 parabolas. This additional effect reflects the positive impact of the parabolas in the absence of both agonists. Our study suggests that acute alterations of gravity significantly increase the BR of hCMs via the LTCC.

## 1. Introduction

It is well established that gravity has had a great impact on the development of all organisms since the emergence of life [1]. Indeed, mechanical stimuli mediated by gravity induce signal transduction pathways, affecting several cellular biological processes [2]. More recently, attention has been focused on the unique medical problems of astronauts. During space flights, astronauts are exposed to several major stress stimuli, including microgravity and radiation. Astronauts exposed to microgravity for several weeks suffer from many health-related complications. Based on the observations of the three Skylab missions of 28, 56, and 84 days, each involving three astronauts, it was concluded that microgravity induced loss of bone density, muscle atrophy, and cardiovascular, hematic, metabolic, and endocrine complications, which are all associated with increased senescence [1,3]. More recently, it has become clear that both microgravity and radiation induce oxidative stress in various living organisms [1,3].

**Figure 1:**
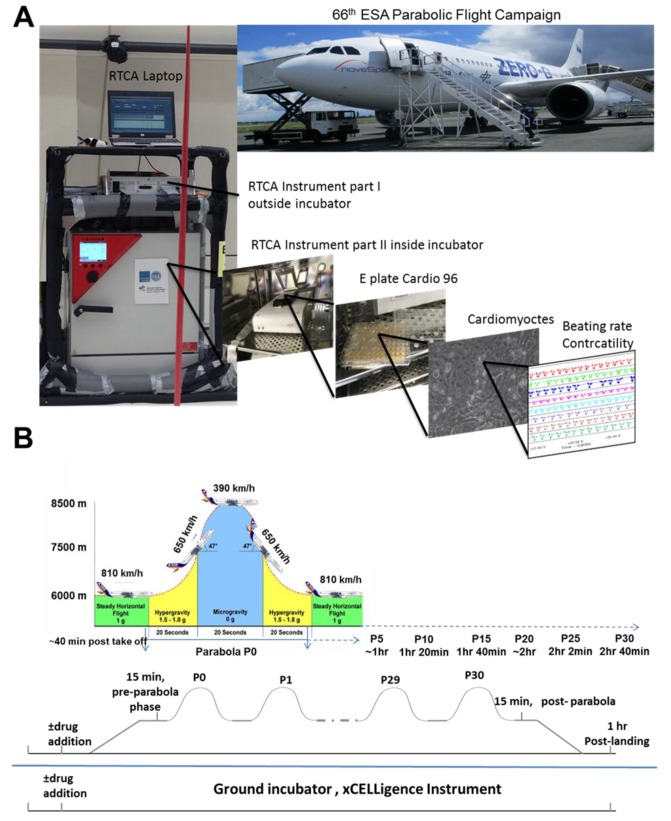
Experimental scheme for parabolic flight experiments carried out during the 66th Parabolic Flight Campaign of the European Space Agency. (**A**) Schematic of the instruments used during parabolic flight experiments. (**B**) Schematic showing flight maneuvers and time points before parabola P0 and after P30.

Notably, the mortality rate of Apollo astronauts was reported to be very high because of heart failure and myocardial infarction problems [4,5]. It is hoped that progress in aerospace medical research will minimize health risks for astronauts. In the long-term, the vision of future space settlement drives several scientific disciplines to investigate the influence of different gravity conditions on human life and health [6]. However, there is a significant gap in developing strategies for fundamental pharmacological interventions for potential microgravity-induced health problems. One of the critical questions that remains to be evaluated is how therapeutics are effective under altered gravity. Fundamental medical pharmaceutical studies under space conditions are cost-intensive and challenging to plan, with very restricted spaceflight opportunities. However, microgravity in the range of 0.01 *g* can be achieved on parabolic flights, offering an alternative means to address pharmaceutical questions, at least under short-term altered gravity conditions (Figure 1). In this scenario, a specially equipped aircraft repeats a parabolic flight path 31 times (completing 31 parabolas), in which each parabola consists of short periods (20–25-s) of 1.8 *g* (hypergravity), ~0.01 *g* (microgravity), and a second 1.8 *g* (hypergravity) period. After completion of the first parabola (P0), a 2-min 1 *g*-recovery period occurs, before starting on subsequent parabolas. In addition, between the 15th and 16th parabola, a 1 *g*-recovery phase of 8 min occurs.

Clearly, human-relevant pharmacological studies require human cellular models to monitor any potential effect of a compound on cellular function. However, there are experimental and ethical limitations to using ex vivo human cells. These limitations can be overcome by investigating functional somatic cell types, derived from human induced pluripotent stem cells (hiPSCs). It is well established that hiPSCs can be randomly differentiated into several somatic cells, including cardiomyocytes [7]. Therefore, hiPSCs offer an unlimited cellular source of functional human cardiomyocytes. We performed fundamental pharmacological studies under different gravity conditions during the parabolic flight campaign by determining the beating rate (BR) of hiPSCs-derived cardiomyocytes (hCMs) in the presence and absence of different agonists, i.e., isoprenalin (ISO), which is a β-adrenoceptor agonist, and Bay-K8644 (Bay-K), which is an L-type Ca^2+^ channel (LTCC) agonist. To measure the BR, we used two identical xCELLigence^®^ real-time cell analyzer (RTCA) Cardio Instruments (ACEA Biosciences, Inc., San Diego, CA, USA), one to monitor cardiomyocyte activity on the ground and another to monitor it during the parabolic flight. Cells were pre-incubated with the active compounds and loaded into both xCELLigence RTCA Cardio Instruments. Observations were carried out for the 3:30-h flight duration to evaluate the effect of repetitive cycles of acute hyper- and micro-gravity. Such fundamental and human-relevant experiments are urgently needed to determine the mechanisms of pharmacological agents targeting cardiovascular receptors and channels under altered gravity conditions, and to determine how microgravity affects cardiac functions. These studies contribute to the development of preventive strategies for astronauts.

## 2. Materials and Methods

### 2.1. Cardiomyocyte Cell Culture

Purified human iCell Cardiomyocytes^®^ (Cellular Dynamics International, Madison, WI, USA), derived from hiPSCs, were used for all experiments. The cardiomyocytes were supplied as a cryopreserved single cell suspension with a ~98% pure population, comprising a mixture of electrically active, atrial-, nodal-, and ventricular-like myocytes. These cells exhibited the typical biochemical, electrophysiological, and mechanical characteristics of normal human heart cells, with expected responses on exposure to exogenous agents. For functional studies, cryopreserved hiPSC-CMs were thawed in iCell Cardiomyocyte Plating Medium (iCell-PM; Cellular Dynamics International, Madison, WI, USA) and directly plated on a fibronectin-coated (5 μg/cm^2^, 2 h at 37 °C) E-plate Cardio 96 (ACEA Biosciences, Inc.) at a cell density of approximately 25 × 10^3^ cells per well, using iCell-PM. For other studies, thawed cells were plated on fibronectin-coated (5 μg/cm^2^, 2 h at 37 °C) 6-well or 96-well plates at a density of 25 × 10^3^ cells per well, respectively. From Day 2 onwards, cells were maintained in iCell Cardiomyocyte Maintenance Medium (iCell-MM; Cellular Dynamics International), with a fresh medium change every 2 days. We observed beating activity 48 h after cell seeding. The hCMs were cultured in a standard cell culture incubator at 5% CO_2_ and 37 °C. Cardiomyocytes cultured for 48 h were treated with ISO (1 µM; Sigma-Aldrich, Corp., St. Louis, MO, USA), LTCC agonist Bay-K (5 μM; Sigma-Aldrich, Corp.), or LTCC blocker nifedipine (1 μM; Sigma-Aldrich, Corp.). These chemicals were added ~40 min prior to performing the parabolic flight experiments.

### 2.2. The xCELLigence RTCA Cardio Instrument

The xCELLigence RTCA Cardio Instrument is an impedance-based platform for monitoring the real-time beating function of the cardiomyocytes [8]. The E-plate Cardio 96 (ACEA Biosciences, Inc.) xCELLigence plates were equilibrated using iCell-PM (50 μL per well) and inserted into the xCELLigence station to measure background impedance and to ensure that all wells and connections were working within acceptable limits. After equilibration, the cells were harvested and seeded at the required density. Impedance measurements were monitored at regular time intervals. The growth area on the E-plate Cardio 96 related to cell adhesion was defined as the Cell Index (CI). A high CI indicated more cell adhesion and higher cell viability. The raw data and statistical information, such as mean and standard error of the mean (SEM) for CI and BR values were acquired using RTCA Cardio software version 1.0 (ACEA Biosciences, Inc.).

### 2.3. Experimental Design

The parabolic flight experiments were performed using the Airbus A310 Zero-G (Novespace, Bordeaux Mérignac Airport, France) during the 66th Parabolic Flight Campaign of the European Space Agency. The experimental hardware consisted of a parabolic flight rack, including a normal cell culture incubator (CB 060; Binder, Tuttlingen, Germany) (Figure 1A). The xCELLigence RTCA Cardio Instrument containing the hCMs was placed within the incubator. Changes in the BR of the hCMs were monitored with a laptop, using the RTCA Cardio software version 1.0 [9]. A typical parabolic flight experiment comprised 31 parabolas (Figure 1B), with alternating acceleration levels of regular gravity (1 *g*). Gravity conditions consisted of an approximately 22-s hypergravity phase (1.8 *g*), a 22-s microgravity phase (~0.01 *g*), followed by another 22-s hypergravity phase (1.8 *g*). The first maneuver of the Airbus A310 Zero-G to create a parabola always counts as the P0, while subsequent maneuvers are recorded as P1 to P30. One campaign consists of three consecutive flight days, with 31 parabolas carried out on each day. Ground experiments were carried out in parallel using a second xCELLigence RTCA Cardio Instrument.

### 2.4. Statistical Analysis

Our statistical analysis was based on Student’s t-test (*p* ≤ 0.05).

## 3. Results

### Beating Rate and Integrity of Human Cardiomyocytes Exposed to Different Gravity Phases

**Figure 2:**
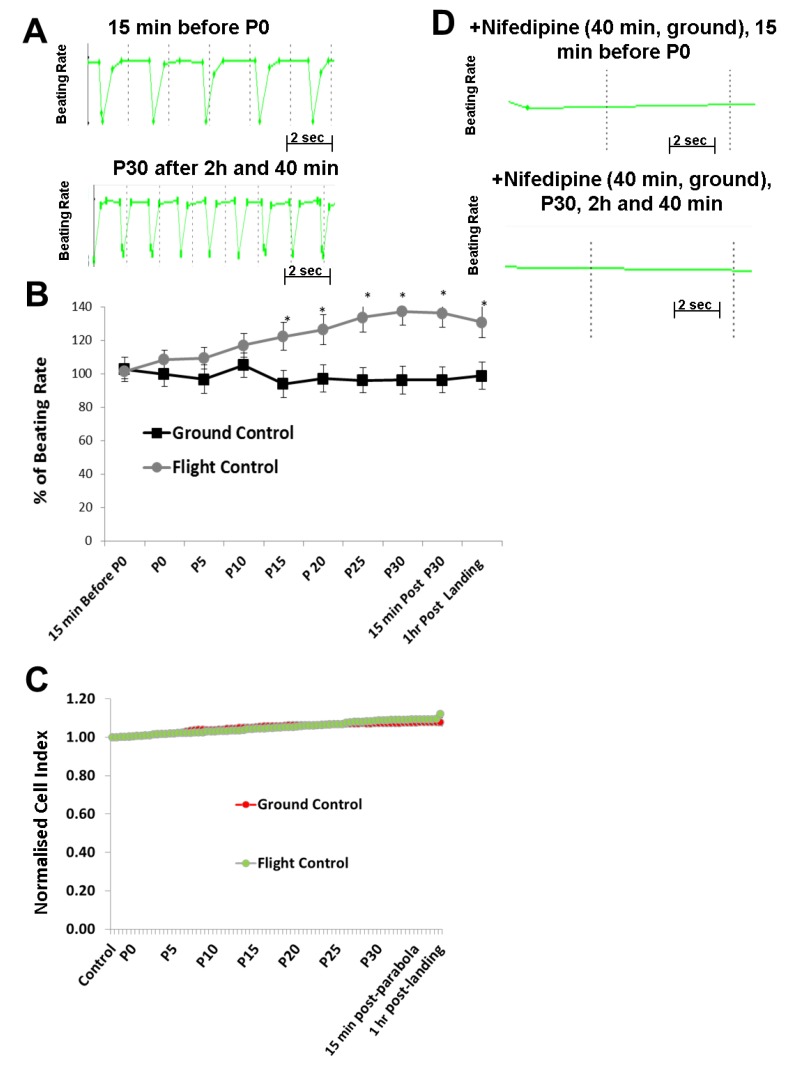
Analysis of the beating rate (BR) of human cardiomyocytes (hCMs) during parabolic flights. (**A**) Representative BR pattern of hCMs at a timepoint 15 min (1 *g*) before starting the first parabola (P0) and after 31 parabolas (P30). (**B**) Data from three independent parabolic flight experiments (each executed in triplicate), as well as data collected from the 1 *g* ground experiment carried out in parallel. The BR values were normalized by setting the control pre-parabola flight or the ground control value to 100% (mean ± SEM; n = 9, * *p* < 0.05 for control versus parabolic flight experiment values). (**C**) Parallel monitoring of the cell viability expressed as cell index (CI) values. The CI values were normalized by setting the control values to 1. (**D**) Representative BR patterns of hCMs treated on the ground (1 *g*) with nifedipine (1 µM) 40 min before starting P0 and after P30. In this case, no BR was observed before P0 or after P30 because of complete inhibition of the L-Type Ca^2+^ Channel. SEM, standard error of the mean.

The CI represents the viability of hMCs, while BR represents their functionality. The parallel sets of ground and flight data for the CI and BR were analyzed to assess changes in the hCMs functional properties during the 31 parabolas. Representative BR traces of non-treated hCMs under 1 *g* flight conditions were recorded 15 min before starting the P0 parabola and after the P30 parabola (Figure 2A). These control traces indicated that an increase in BR occurred on all three independent parabolic flights executed on different days. Each experiment was performed in triplicate on a given day, yielding nine results for each experimental condition (n = 9). Values in the absence of agonists were normalized to 1 *g* ground control values to yield percentages. As indicated in Figure 2B, the BR of the hMCs (in the absence of any agonist or antagonist) rose as the number of parabolas increased, showing a maximal increase of 40% after P30. Post-landing (1 *g*), the BR decreased again (Figure 2B). In contrast, the CI remained stable during the parabolic flights (Figure 2C), suggesting that the different gravity conditions had no effect on the viability and integrity of the hCMs. To determine whether the increase in beating activity during the parabolic flight was mediated by the LTCC, we pre-incubated the cells with the LTCC blocker nifedipine (1 µM) for 40 min prior to parabola P0. In the presence of nifedipine, the BR was completely blocked, even after 31 parabolas (Figure 2D).

**Figure 3:**
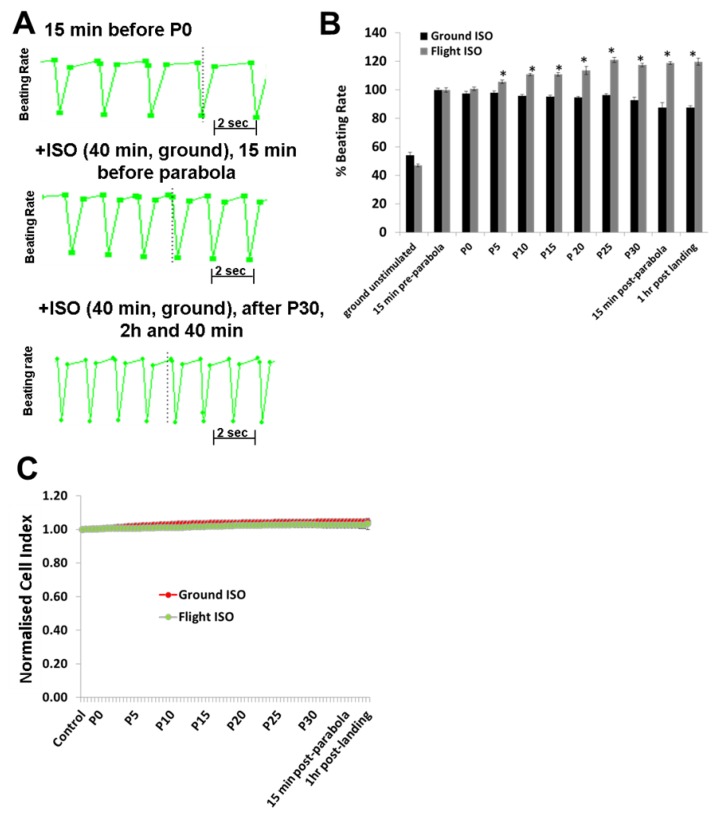
Analysis of the beating rate (BR) of human cardiomyocytes (hCMs) stimulated with isoprenalin (ISO; 1 µM) during the parabolic flight experiments (**A**) **Upper panel:** Representative BR pattern of unstimulated hCMs recorded 15 min (1 *g*) prior to the first parabola (P0); **Middle panel:** Representative BR pattern of hCMs when stimulated with agonists 40 min prior to the parabolic flight and monitored 15 min before starting parabola P0. **Lower panel:** Representative BR pattern of hCMs measured after 31 parabolas. (**B**) Data from three independent parabolic flight experiments, each executed in triplicate, as well as data collected from the 1 *g* ground experiment carried out in parallel. The results were normalized by setting the ISO-induced BR of the ground control experiment prior to P0 to 100% (mean ± SEM, n = 9, * *p* < 0.05 for the ISO control versus the ISO parabolic flight values). (**C**) Parallel monitoring of the cell viability expressed as the cell index (CI) value. The CI values were normalized by setting the control values to 1. SEM, standard error of the mean.

**Figure 4:**
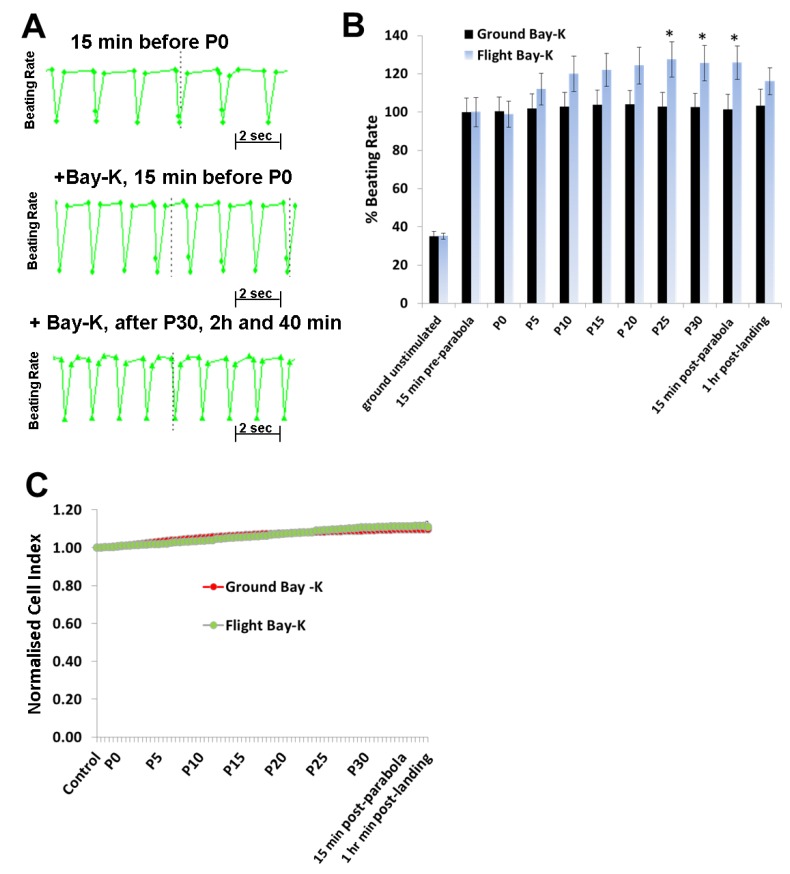
Analysis of the beating rate (BR) of human cardiomyocytes (hCMs) stimulated with Bay-K8644 (Bay-K; 1 µM) during the parabolic flights experiments (**A**) **Upper panel:** Representative BR pattern of unstimulated hCMs recorded 15 min (1 *g*) prior to the first parabola (P0); **Middle panel:** Representative BR pattern of hCMs when stimulated with agonists 40 min prior to P0 and monitored 15 min before starting parabola P0. **Lower panel:** Representative BR pattern of hCMs stimulated 40 min prior to P0 and monitored again after 31 parabolas. (**B**) Data from three independent parabolic flight experiments, each executed in triplicate, as well as data collected from 1 *g* ground experiments carried out in parallel. The BR values were normalized by setting the Bay-K-induced BR of the ground control experiments prior to P0 to 100% (mean ± SEM, n = 9, * *p* < 0.05 for the ISO control values versus the Bay-K parabolic flight values). (**C**) Parallel monitoring of the cell viability expressed as the cell index (CI). The CI values were normalized by setting the control values to 1. SEM, standard error of the mean.

Both ISO (1 µM) (Figure 3B) and Bay-K (1 µM) (Figure 4B) increased the BR of the hCMs when they were stimulated under 1 *g* ground conditions (40 min before starting P0). Compared to the BR of hCMs recorded during the flight control in the absence of the agonists (Figure 3A, Figure 4A; upper panels), in the presence of agonists, the BR of the hCMs was higher (Figure 3A, Figure 4A; middle panels) and became even higher after 31 parabolas (after 2:40 h) (Figure 3A, Figure 4A; lower panels). The agonist-induced elevation in BR of the hCMs was statistically validated by normalizing their values against the values of the 1 *g* ground samples treated with ISO (1 µM) or Bay-K (1 µM) stimulation, which were set to 100% (n = 9). Cardiomyocytes which were prestimulated with ISO or Bay-K on the ground showed twofold and 2.5-fold BR increases, respectively, compared with their 1 *g* ground stimulated samples (ISO: Figure 3B; Bay-K: Figure 4B). Interestingly, after the fifth parabola (P4), an additional effect on ISO or Bay-K8644 stimulated samples was observed, proportional to the increasing number of the parabolas (post P24) and showing a maximal additional effect of 20% and 25%, respectively (ISO: Figure 3B; Bay-K: Figure 4B). The maximal additional effect for both agonists remained stable for at least 1 h post-landing. In contrast, the CI values remained stable during all parabolic flight experiments, suggesting that these gravity conditions had no effect on the viability and integrity of hCMs (Figure 3C and Figure 4C).

## 4. Discussion

Recently, embryonic stem cells have been used to study the effects of different small signaling molecules [10] and microgravity [11] on the embryonic development processes. To do this, we established in vitro methodologies allowing differentiation of mouse embryonic stem cells under simulated microgravity within a fast-rotating clinostat (clinorotation) [12] and under parabolic flight conditions [11]. Using these methods, we identified developmental processes affected by microgravity using microarray technologies. To determine the mechanisms of cardiovascular-acting drugs under different gravity conditions, hCMs were exposed to alternate short periods of hypergravity (22-s), microgravity (22-s), and hypergravity (22-s) during a single parabolic loop for 31 parabolas. During exposure of the hCMs to this increasing number of parabolas, the BR and CI, representing hCM functionality and viability, were monitored using the xCELLigence RTCA Cardio Instrument. In parallel, corresponding control experiments were carried out on the ground (1 *g*) using the same experimental set up. Interestingly, our results indicated that the BR of hCMs was significantly accelerated as the number of parabolic cycles increased. However, the Cl values were not affected, demonstrating that the mechanical stress did not modify hCMs viability. The BR was mediated by the LTCC, allowing an influx of extracellular Ca^2+^ which normally binds to the ryanodine receptor of the sarcoplasmic reticulum, thereby inducing a massive Ca^2+^ release from the sarcoplasmic reticulum, a process known as the Ca^2+^-induced Ca^2+^-release mechanism. In another set of the experiments, we preincubated the hCMs with nifedipine prior to the parabolic flight experiment. As expected, prior to lift off, nifedipine completely blocked the beating of the hCMs. Notably, no beating activity was observed during the parabolic flight in the presence of nifedipine, even after 31 parabolas. This finding clearly indicates that the elevation of the BR observed with an increasing number of parabolas was mediated by the LTCC. We surmise that the mechanical stress induced by the high number of parabolas increased the activity of the LTCC. This is supported by the observation that mechanical stress modulates the stability and activity of cardiomyocytes, e.g., acute mechanical stress induces opening of the LTCC [13]. Mechanical stress also appears to induce elevation of cytosolic Ca^2+^ in rat cardiomyocytes, mediated by the LTCC via the Ca^2+^-induced Ca^2+^-release mechanism [14]. Therefore, we postulate that the mechanical stress induced by parabolic-flight-altered gravity conditions initiated an increase in BR via activation of the LTCC, although the detailed mechanism is yet to be determined.

Recently, it was demonstrated that gravity impacts on membrane fluidity, raising new ideas about how cells might experience gravity [15]. Clearly, altered gravity (during parabolic flight conditions) alters membrane fluidity, voltage-gated K^+^ channel activity, and signal transduction pathways mediated by some transmembrane proteins (e.g., receptors) [16]. It is also well known that changes in membrane fluidity of the sarcoplasmic reticulum affect the activity of the ryanodine receptor, which releases Ca^2+^ from the sarcoplasmic reticulum that regulates the BR of cardiomyocytes [17]. Gravity-related changes in cell membranes are a very interesting aspect, but they do not provide an explanation for distinct changes in the activity for a specific channel, as observed herein for the LTCC in hCMs.

To investigate the effects of hyper- and micro-gravity on the interactions of ISO with the β-adrenoceptor of the cardiomyocytes and of Bay-K with the LTCC, we prestimulated the hCMs with agonists and exposed them to parabolic flight conditions. Both ISO and Bay-K increased the BR of the hCMs under 1 *g* ground control and pre-parabola conditions. However, we observed an additional increase of about 20% after 25 parabolas during the experiments. We assume that this additional effect is related to mechanical stress accumulated after 25 parabolas. We also investigated the effect of Carbachol (1 µM) on the BR under 1 *g* conditions. However, we did not observe any effects on the basal BR, although carbachol mitigated the additional effect of ISO (data not shown). Similar results with Carbachol were described recently [18].

In conclusion, alternate hypergravity and microgravity phases increased the BR of hCMs, most probably mediated by the LTCC. Moreover, alternate hypergravity and microgravity phases had no effects on the interactions of agonists with the β-adrenoceptor or the LTCC, given that we only observed an additional stimulating effect of approximately 20%, which could be induced by mechanical stress during the parabola flight conditions.

Several experiments have been conducted with subjects exposed to parabolic flights. Standing subjects exposed to parabolic flight conditions indicated an increase of 12 to 16 heart beats per minute (bpm) within the first 1.8 *g* phase, a decrease of 27 to 15 bpm within the microgravity phase (0.01 *g*) and an increase of 20 to 24 bpm during the second 1.8 *g* phase [19,20,21]. Interestingly, using the electrocardiography (ECG) method for determining the heart rate, an increase in the bpm was observed after completion of the 15 parabolas, with a maximum effect after completion of five parabolas [19]. This study demonstrated that, although there were fluctuations in the bpm within the three phases of one parabolic cycle, the mean bpm was maximally increased after completion of five parabolas [19]. These results can be explained by an activation of the sympathetic and competing parasympathetic neuronal systems during the parabolic flight, with a higher activity of the sympathetic system after at least 15 parabolas. Indeed, the in vivo neuronal mechanisms regulating the beating activity of the heart are completely absent in our in vitro system. However, although we did not observe differences in the BR over the three different phases of each parabola (supplementary Figure S1), we found that the increase in the BR of hCMs was proportional to the number of the parabolas completed.

A clear understanding of the effects of microgravity on cardiomyocyte functions may only be obtained by culturing them under long-term (several weeks) microgravity conditions, which would need to be performed on the International Space Station. We believe that our pilot work under parabolic flight conditions provides worthwhile data for the design of experiments under a long-term microgravity environment. Such experiments would contribute to our ongoing understanding of the underlying molecular mechanisms of gravity-induced cardiomyocyte altered functioning and to the development of preventive pharmacological applications for microgravity-associated cardiovascular diseases.

## Supplementary Materials

The following are available online at https://www.mdpi.com/2073-4409/8/4/352/s1, Figure S1: Effects of hypergravity, microgravity and hypergravity within an individual parabola.
