# Supplementary material for: Parabolic, Flight-Induced, Acute Hypergravity and Microgravity Effects on the Beating Rate of Human Cardiomyocytes
Source: Cells. 2019 Apr 14;8(4):352. doi: 10.3390/cells8040352 (PMC6523861; doi:10.3390/cells8040352)
Supplement: Supplementary file 1 [file cells-08-00352-s001.pdf]

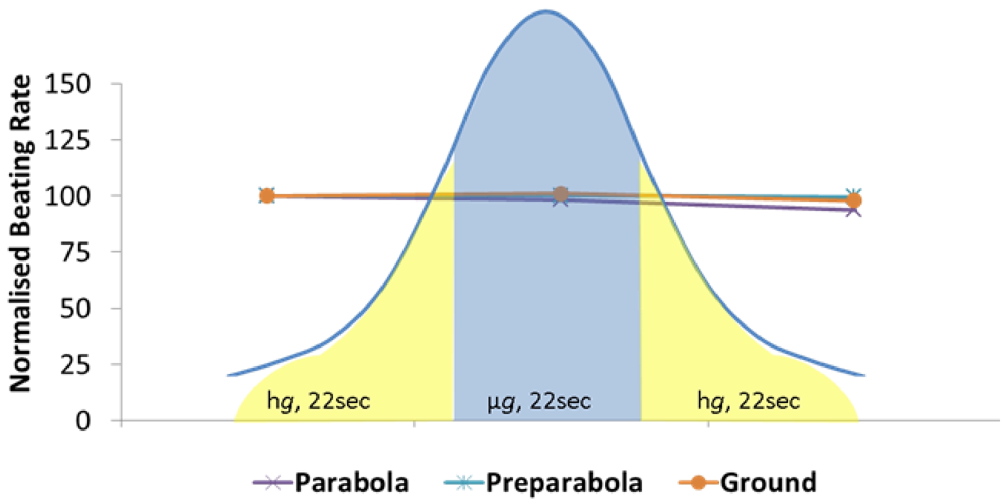

Figure S1: Effects of hypergravity, microgravity and hypergravity within an individual parabola. No differences in the beating rate was observed in any of the individual 31 parabolas among phases of hypergravity and microgravity. All data from the three separate flight days were normalized to the values of the 1 *g* flight control.
